# Corneal Astigmatism Outcomes After Simultaneous Pterygium Excision Using The Bare Sclera Technique and Manual Small Incision Cataract Surgery: Protocol for a Prospective Single-Arm Pre-Post Interventional Cohort Study

**DOI:** 10.2196/96749

**Published:** 2026-09-23

**Authors:** Akshaya Tandle, Kanchan Selukar

**Affiliations:** 1 Department of Ophthalmology Jawaharlal Nehru Medical Science Datta Meghe Institute of Higher Education and Research Wardha, Maharashtra India

**Keywords:** pterygium, cataract, small incision cataract surgery, corneal astigmatism, Alpins vector analysis, keratometry

## Abstract

**Background:**

Pterygium and cataract frequently coexist in populations with high UV light exposure, particularly in rural India. Both conditions impair visual acuity and reduce quality of life. Simultaneous small incision cataract surgery (SICS) combined with pterygium excision offers the advantages of a single surgical session, faster rehabilitation, and lower cost compared with sequential procedures. However, SICS uses a superior scleral tunnel incision of 6.5 to 7 mm, which itself induces against-the-rule surgically induced astigmatism. The net astigmatic outcome of simultaneous SICS and pterygium excision—specifically, whether the reduction of pterygium-induced with-the-rule astigmatism offsets SICS-induced against-the-rule astigmatism—has not been prospectively characterized in a resource-limited setting. The bare sclera technique, while not designed to minimize recurrence, was selected to isolate the corneal astigmatic effects of fibrovascular tissue removal without confounding variables associated with conjunctival grafting.

**Objective:**

This protocol describes a prospective single-arm pre-post interventional cohort study designed to quantify the change in the magnitude and axis of corneal astigmatism from baseline to day 30 following manual SICS with posterior chamber intraocular lens (IOL) implantation, followed by primary nasal pterygium excision using the bare sclera technique, in a single operative sitting.

**Methods:**

This prospective single-arm pre-post interventional cohort study will be conducted at the Department of Ophthalmology, Acharya Vinoba Bhave Rural Hospital, Datta Meghe Institute of Higher Education and Research (DMIHER), Wardha, India. A total of 100 participants aged ≥35 years with visually significant cataract and grade 1 to 2 primary nasal pterygium will undergo standardized simultaneous surgery consisting of manual SICS with posterior chamber IOL implantation, followed by bare sclera pterygium excision in a single operative sitting. The study evaluates short-term postoperative outcomes over 30 days; long-term corneal stabilization and recurrence assessment are beyond its scope. As this is a single-arm exploratory study without a comparator group, causal inferences will not be made. The sample size (N=100) was calculated using paired continuous outcomes based on the expected change in keratometric astigmatism. The primary outcome is change in keratometric corneal astigmatism (magnitude and axis) from baseline to day 30. Secondary outcomes include Alpins vector analysis, the association between pterygium size and preoperative astigmatism, changes in visual acuity, refractive surprise, early fibrovascular regrowth, patient satisfaction, and complications. Follow-up assessments will occur on day 1, day 15, and day 30.

**Results:**

Ethics approval was obtained on June 30, 2025. Participant recruitment began on November 1, 2025. As of September 2026, 40 participants have been enrolled. Data analysis is expected to be conducted following completion of follow-up.

**Conclusions:**

This protocol will generate prospective data on corneal astigmatism following simultaneous manual SICS and bare sclera pterygium excision to inform surgical planning, IOL selection, and patient counseling in SICS-predominant resource-limited settings.

**Trial Registration:**

Clinical Trials Registry–India CTRI/2025/10/095785; https://tinyurl.com/mry8se6y

**International Registered Report Identifier (IRRID):**

DERR1-10.2196/96749

## Introduction

### Background and Rationale

Pterygium is a fibrovascular proliferation of the bulbar conjunctiva that encroaches onto the corneal surface in a wing-shaped configuration, with the highest prevalence in equatorial regions between latitudes 37° and 40° north and south [1,2]. Recent global prevalence estimates indicate that pterygium affects approximately 10% to 15% of adults in tropical and subtropical regions, with higher rates in outdoor occupational groups [3]. Chronic UV radiation exposure and advancing age are linked to the development of pterygium [4]. Cataract also shares UV radiation as a primary causative factor, and both conditions frequently coexist in older populations of low- and middle-income nations such as India [4,5]. Currently, >10% of India’s population is aged ≥60 years, and this proportion is projected to reach 15% by 2036. This amplifies the burden of pterygium (prevalence 13.2% among rural adults aged ≥40 years) and cataract (58%-72% in the older adult population), both of which impose substantial visual morbidity and economic costs [4,6,7]. According to the National Blindness and Visual Impairment Survey (2019-2020), cataract is India’s leading cause of blindness, while pterygium is a major contributor to corneal astigmatism in rural populations [8,9].

Pterygium flattens the corneal surface focally along the horizontal meridian, thereby inducing corneal astigmatism. Its severity is directly proportional to the length, width, and depth of corneal encroachment [10,11]. Even before the visual axis is threatened, pterygium-induced with-the-rule astigmatism can substantially degrade uncorrected visual acuity (UCVA). Furthermore, pterygium distorts manual keratometry readings, producing artificially low corneal curvature values and leading to systematic errors in intraocular lens (IOL) power calculation [12,13].

When pterygium and cataract coexist, sequential surgery (excision first, followed by cataract surgery after stabilization) yields more predictable refractive outcomes, as IOL power is calculated on a pterygium-free cornea [14,15]. However, sequential surgery imposes a disproportionate burden on patients in rural settings who cannot attend multiple surgical admissions. Simultaneous surgery is therefore commonly practiced in tertiary care centers serving such populations [14].

Manual small incision cataract surgery (SICS) predominates in high-volume rural India, as it requires no expensive ultrasound and yields consistently good outcomes. Recent large-scale studies from India have reported excellent visual outcomes following SICS, with more than 90% of patients achieving good postoperative visual acuity [16,17]. However, SICS-induced astigmatism remains a significant consideration, with reported against-the-rule shifts ranging from 0.5 to 1.5 D depending on incision size and location [18]. Simultaneous pterygium excision and phacoemulsification with IOL implantation significantly reduces astigmatism (eg, from 1.98 D to 0.54 D), but phacoemulsification is scarce in resource-limited settings [9,19].

Prospective studies specifically evaluating corneal astigmatism outcomes following simultaneous pterygium excision combined with manual SICS remain limited in the published literature. This gap is clinically significant because the larger scleral tunnel of SICS (6.5-7 mm) introduces a competing against-the-rule astigmatic vector that may substantially alter the net refractive outcome compared with that of phacoemulsification-based simultaneous surgery. Without prospective SICS-specific data, clinicians in SICS-predominant settings lack evidence on which to base surgical planning, IOL power selection, and patient counseling.

### Objectives

#### Primary Objective

The primary objective is to quantify the change in the magnitude and axis of corneal astigmatism (keratometric cylinder, measured in diopters and degrees) from baseline to day 30 following a protocol-defined simultaneous surgical intervention consisting of manual SICS with posterior chamber IOL implantation and primary nasal pterygium excision using the bare sclera technique in a single operative sitting.

#### Secondary Objectives

The secondary objectives are to characterize the temporal trajectory of astigmatism change at day 1, day 15, and day 30; to assess surgically induced astigmatism (SIA) using Alpins vector analysis; to evaluate the correlation between pterygium grade, length, and width and the magnitude of preoperative corneal astigmatism; to quantify changes in uncorrected and best-corrected visual acuity (BCVA) across all follow-up time points; to determine the rate and magnitude of refractive surprise and its continuous relationship with pterygium size using regression analysis; to assess patient-reported satisfaction with visual outcome at day 30 using a pilot-tested structured questionnaire; and to document intraoperative and postoperative complications.

## Methods

### Study Design, Study Setting, and Duration

This prospective single-arm pre-post interventional cohort study will be conducted at the Department of Ophthalmology, Acharya Vinoba Bhave Rural Hospital (AVBRH), Maharashtra, India. The intervention consists of a protocol-defined simultaneous surgical procedure: manual SICS with posterior chamber IOL implantation followed by primary nasal pterygium excision using the bare sclera technique, performed in a single operative sitting under peribulbar anesthesia. All enrolled patients will receive this standardized intervention as per protocol; the surgical sequence and technique will be uniform across all cases. This study is classified as interventional by virtue of the protocol-defined standardized surgical procedure. Preoperative measurements from each enrolled participant serve as the within-patient comparator for all postoperative time points. There is no external comparison group; findings will be interpreted against the existing phacoemulsification-based comparative literature. No formal data monitoring committee was constituted due to the low-risk nature of the intervention.

The estimated study duration is 2 years, with a 30-day follow-up to assess early postoperative astigmatic outcomes; long-term stability and recurrence are beyond the scope of the study.

### Sample Size Calculation

Sample size was calculated using the primary outcome: change in keratometric corneal astigmatism (diopters) from baseline to postoperative day 30. Because the primary end point is a paired continuous outcome measured repeatedly in the same eye, sample size estimation based on paired mean differences was considered methodologically appropriate.

Sample size for paired continuous outcomes was calculated using the following formula:



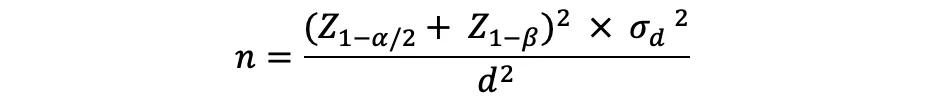



where

- Z_1−α/2_=1.96 (for α=.05, 2-sided)
- Z_1−β_=1.28 (for 90% power)
- σ_d_=expected SD of the paired differences in astigmatism
- d=clinically meaningful difference (minimal clinically important difference [MCID])

Under assumptions of α=.05 (2-sided), 90% power, an exploratory clinically meaningful difference (MCID) of 0.75 D (selected as an exploratory clinically relevant change based on cataract surgery literature in the absence of a procedure-specific MCID), and an SD of paired differences (σ_d_) of 1.5 D, the calculated minimum sample size was approximately 65 participants. To ensure adequate power for secondary analyses, including Alpins vector analysis and regression modeling, the target sample size was increased to 72 participants. After allowing 20% loss to follow-up, the final sample size was set at 100 participants.

### Eligibility Criteria

#### Inclusion Criteria

Participants aged ≥35 years of either sex with clinically diagnosed visually significant cataract (nuclear sclerosis grade ≥2 by Lens Opacities Classification System III [LOCS-III]) and concurrent primary nasal pterygium in the same eye will be eligible. Eligible pterygia include grade 1 (<2 mm corneal encroachment from the limbus) or grade 2 (2-4 mm corneal encroachment), measured using a calibrated slit-lamp graticule according to the classifications described by Kodavoor et al [9] and Tan et al [20]. Participants must be willing and able to comply with follow-up visits on day 1, day 15, and day 30 and provide written informed consent in English, Hindi, or Marathi.

#### Exclusion Criteria

Participants with grade 3 (>4 mm corneal encroachment) or grade 4 pterygium; recurrent, temporal, or double-headed pterygium; traumatic, developmental, or congenital cataract; previous ocular surgery; or coexisting ocular pathology likely to affect visual or refractive outcomes will be excluded. Additional exclusion criteria include poor pupillary dilation (<6 mm); recent contact lens wear that may influence keratometric measurements (rigid lenses within 4 weeks or soft lenses within 72 hours); uncontrolled systemic disease, including diabetes mellitus (random blood glucose >200 mg/100 mL) or hypertension (>160/100 mm Hg); and known bleeding disorders.

### Recruitment and Screening

Participants will be recruited from patients at the ophthalmology outpatient department (OPD), those attending camps, and those admitted for elective cataract surgery at AVBRH who have concurrent pterygium identified on routine slit-lamp examination. The principal investigator will assess all potentially eligible patients and obtain informed consent before any study procedures. A prospective screening log will document screening, eligibility, exclusions, and reasons for nonenrollment.

### Preoperative Assessment

All eligible participants will undergo a standardized preoperative assessment, including UCVA and BCVA (Snellen charts at 6 meters, converted to logarithm of the minimum angle of resolution [logMAR]), manifest refraction (autorefractometer, Rexxam Accuref KR-800 (Rexxam Co Ltd) or equivalent), slit-lamp examination with cataract grading (LOCS-III), and quantitative assessment of pterygium morphology (grade, length from the limbus to the apex, and width at the limbal base using a calibrated graticule). Corneal keratometry (manual Javal-Schiotz or automated) will be performed by a masked examiner using the mean of 3 readings. Recorded parameters will include K1, K2, principal axis, and corneal cylinder. Corneal topography will not be routinely performed, reflecting standard practice in this high-volume rural setting. Although previous studies have suggested that 3D parameters, such as pterygium thickness, may correlate with astigmatism, advanced imaging modalities required for these assessments are not routinely available in the study setting. Therefore, clinically feasible morphological parameters, including grade, length, and width, were selected. Additional assessments will include dilated posterior segment examination, axial length measurement by contact A-scan biometry, intraocular pressure by noncontact tonometry, and systemic evaluation, including blood pressure, pulse rate, temperature, and random blood glucose.

### IOL Power Calculation and Refractive Surprise Analysis

Pterygium-induced corneal flattening causes underestimation of keratometry values, leading to IOL power overestimation and a predictable postoperative myopic shift after pterygium excision [14,21]. Kamiya et al [21] demonstrated, in the context of phacoemulsification-based simultaneous surgery, that the magnitude of this myopic shift correlates with pterygium size; however, no empirically validated fixed correction formula exists for simultaneous surgery in the SICS context [14].

Given this, the following transparent and exploratory approach will be adopted: IOL power will be calculated using the Sanders Retzlaff Kraff theoretical (SRK/T) formula targeting emmetropia, with axial length measured by A-scan ultrasonography and corneal power obtained from automated keratometry. No empirical adjustment of IOL power will be applied, as no validated correction formula currently exists for simultaneous SICS-pterygium surgery. Postoperative refractive surprise, defined as the achieved postoperative spherical equivalent minus the predicted postoperative spherical equivalent derived from IOL power calculation, will be assessed at day 30; deviations greater than 1 D will be considered clinically significant. Linear regression analysis will evaluate pterygium size (continuous, in mm from the limbus) as a predictor of refractive surprise magnitude. Additionally, exploratory sensitivity analyses will compare refractive surprise rates among participants divided into pterygium size tertiles: <2.5 mm, 2.5 to 3.5 mm, and >3.5 mm.

### Surgical Procedure and Quality Assurance

All procedures will be performed by a single surgeon under guided supervision using peribulbar anesthesia (2% lignocaine and 0.5% bupivacaine with hyaluronidase). Quality assurance will comprise early recruitment supervision and incision dimension documentation for exploratory analyses.

### Surgical Sequence

Manual SICS will always be carried out first to preserve globe stability and avoid ocular surface hemorrhage that can obstruct cataract extraction. After IOL implantation, the pterygium head will be carefully dissected from the cornea using a surgical blade, and fibrovascular tissue will be excised from the underlying scleral bed using blunt and sharp dissection. The exposed scleral area will be left uncovered (bare sclera technique), without conjunctival autograft or suturing. Although autografting has lower recurrence rates, the bare sclera technique was selected because the primary objective is to characterize changes in corneal astigmatism rather than to prevent pterygium recurrence. During follow-up, detailed documentation of early fibrovascular regrowth (fibrovascular tissue reaching >1 mm from the limbus onto the cornea) will be made. Definitive assessment of pterygium recurrence is beyond the scope of the 30-day follow-up period. Hemostasis will be achieved with gentle bipolar cautery where required.

### SICS Procedure

A self-sealing triplanar superior scleral tunnel incision, centered at 12 o’clock, will be fashioned at a width of 6.5 to 7 mm. An anterior capsulorhexis will be performed where feasible; a can-opener capsulotomy will be used if pupil dilation is suboptimal. Nucleus delivery will be performed by manual expression. Cortical wash will be performed using a Simcoe irrigation-aspiration cannula. A rigid PMMA (poly [methyl methacrylate]) posterior chamber IOL of appropriate power will be implanted in the capsular bag. Wound sutures will be placed only if self-sealing integrity is inadequate. Subconjunctival dexamethasone and gentamicin will be administered at the close of surgery. Intraoperative complications (posterior capsular rent, iris prolapse, zonular dialysis, and vitreous loss) will be documented contemporaneously.

### Postoperative Regimen and Assessment

All participants will receive topical prednisolone acetate 1% and moxifloxacin combination (tapering from once hourly to 4 times daily over 4 weeks), topical moxifloxacin 0.5% (4 times daily for 2 weeks), and lubricating eye drops as required. Eye protection instructions will be provided in the participant’s preferred language.

All participants will undergo a standardized assessment at each time point according to the schedule outlined in Table 1.

**Table 1:** Schedule of assessments across all study visits, including preoperative, intraoperative, and postoperative data collection at baseline and on days 1, 15, and 30 (primary end point visit).

Assessments	Baseline	Intraoperative	Day 1	Day 15	Day 30
Uncorrected visual acuity (Snellen, converted to logMAR^a^)	✓			✓	✓
Best-corrected visual acuity (Snellen, converted to logMAR)	✓			✓	✓
Manifest refraction (autorefractometer)	✓			✓	✓
Keratometry: K1, K2, and axis (3 masked readings)	✓		✓	✓	✓
Slit-lamp anterior segment examination	✓	✓	✓	✓	✓
Dilated fundus examination	✓				✓
Intraocular pressure (noncontact tonometry)	✓		✓	✓	✓
A-scan biometry (axial length)	✓				
Corneal fibrovascular regrowth from the limbus (mm)	✓		✓	✓	✓
Incision size (caliper measurement)		✓			
Systemic parameters (blood pressure, pulse, and random blood sugar)	✓				
Intraoperative complications		✓			
Postoperative complications		✓	✓	✓	✓
Patient satisfaction questionnaire					✓

^a^logMAR: logarithm of the minimum angle of resolution.

Because postoperative inflammation may influence keratometric measurements, slit-lamp evidence of clinically significant postoperative inflammation will be documented during follow-up visits and considered during interpretation of outcome measurements. Inflammation will be graded clinically using slit-lamp examination findings (anterior chamber cells, flare, and anterior segment reaction).

### Outcome Measures

The primary outcome is change in corneal astigmatism from baseline to day 30 assessed using keratometric cylinder (diopters), derived from automated keratometry (mean of 3 consecutive readings obtained by a masked examiner). Both magnitude and axis of astigmatism will be recorded. Astigmatism will be reported both as a continuous variable (mean cylindrical error [SD]) and as categories of 0 D, >0 to ≤1 D, >1 to ≤2 D, and >2 D (absolute magnitude). The predefined clinically relevant threshold of 0.75 D will be interpreted as an exploratory benchmark rather than a validated procedure-specific MCID. The net change in keratometric cylinder magnitude and axis will be characterized descriptively and analytically. The primary end point is the change from baseline to day 30; day 1 and day 15 are secondary longitudinal assessments and will not be used for primary hypothesis testing.

The secondary outcomes include (1) the trajectory of astigmatism change on day 1, day 15, and day 30; (2) Alpins vector analysis parameters (target induced astigmatism vector [TIA], SIA, difference vector [DV], correction index, and index of success) calculated using ASSORT (Alpins Statistical System for Ophthalmic Refractive Surgery Techniques; ASSORT Pty Ltd) software [22], and institutional access to ASSORT software was confirmed before study initiation; (3) the correlation between pterygium grade, length, and width and preoperative corneal astigmatism using Spearman ρ; (4) the change in UCVA and BCVA from baseline to each follow-up visit, categorized as 6/6-6/12, 6/18-6/60, or <6/60; (5) refractive surprise, defined as a deviation of >1 D between the achieved and predicted spherical equivalent at day 30, analyzed by continuous regression and tertile subgroup analysis by pterygium size; (6) patient satisfaction at day 30 using a pilot-tested 10-item questionnaire; (7) the incidence and type of intraoperative complications (posterior capsular rent, iris prolapse, zonular dialysis, and vitreous loss); and (8) the incidence and type of postoperative complications, including early fibrovascular extension or regrowth documented as a descriptive postoperative observation.

### Data Collection and Management

Data will be recorded by the principal investigator on a structured case record form (CRF) at each visit (Multimedia Appendix 1). The CRF covers demographic data, systemic parameters, preoperative findings, intraoperative events, and postoperative follow-up data at each time point. Paper CRFs will be stored in a locked research cabinet accessible only to the principal investigator and study guide. Data will be double-entered into Excel (version 2019; Microsoft Corp) and exported to SPSS (version 23.0; IBM Corp). Transcription errors will be checked by random cross-verification of 10% of records. Participant identifiers will be replaced with unique numeric codes in the analysis dataset.

### Patient-Reported Outcome Measure and Pilot Testing

Patient satisfaction at day 30 will be assessed using a pilot-tested investigator-developed 10-item questionnaire administered in English, Hindi, or Marathi. Domains assessed include visual improvement, symptoms, visual function, spectacle dependence, daily activities, and overall satisfaction using 3-point response scales.

Pilot testing in 10 patients demonstrated acceptable internal consistency (Cronbach α=0.76). Minor wording modifications were made following participant feedback. The questionnaire is considered a study-specific assessment tool and not a validated patient-reported outcome measure.

### Statistical Analysis

Descriptive statistics (mean [SD] or median [IQR] for continuous variables; frequencies [percentages] for categorical variables) will be reported. The primary outcome (baseline to day 30 astigmatism change) will be analyzed using a paired *t* test or Wilcoxon signed-rank test (*P*<.05, 2-tailed) among participants who complete both baseline and day 30 assessments. Participants without day 30 measurements will be excluded from the primary analysis; however, they will contribute available data to secondary longitudinal analyses using mixed models for repeated measures (MMRM). The Shapiro-Wilk test will be used to assess normality of paired differences; the paired *t* test will be applied for normally distributed differences, while the Wilcoxon signed-rank test will be used for nonnormal distributions. The paired analysis will compare baseline and day 30 values only, as day 30 represents the predefined primary end point. Longitudinal analyses of day 1, day 15, and day 30 measurements will be performed separately using MMRM with random intercepts. Alpins vector analysis (TIA, SIA, DV, correction index, and index of success) will be performed using ASSORT software. Spearman ρ will assess relationships between pterygium dimensions and preoperative astigmatism, while exploratory univariable regression analysis will evaluate pterygium size as a predictor of refractive surprise magnitude at day 30. Tertile subgroup comparisons of refractive surprise rates will be analyzed using chi-square tests. Secondary analyses will be considered exploratory and interpreted without formal multiplicity adjustment.

Complication rates will be reported as proportions with corresponding 95% CIs calculated using the Wilson method.

### Masking

The outcome assessor performing keratometry and manifest refractions will be masked to pterygium grade and follow-up time point. The surgeon and participant cannot be masked due to the nature of the surgical intervention.

### Adverse Event Reporting

All intraoperative and postoperative complications will be documented in the CRF. Serious adverse events will be reported according to the institutional ethics committee requirements. A complete list of expected and unexpected adverse events is maintained in the study master file.

### Missing Data

The primary end point is change in keratometric corneal astigmatism from baseline to day 30. The primary analysis will therefore use a complete-case approach, including participants with both baseline and day 30 measurements. The extent, pattern, and reasons for missing data will be reported. If missing day 30 data exceed 10%, sensitivity analyses using MMRM will be emphasized.

For secondary longitudinal analyses across day 1, day 15, and day 30, MMRM will be used to incorporate participants with incomplete follow-up under a missing-at-random assumption without formal imputation. The model will include time as a categorical fixed effect and participant as a random effect.

As a sensitivity analysis, MMRM-derived estimates of day 30 astigmatism change will be compared with the complete-case primary analysis to assess the impact of missing data.

### Bias and Confounding

Potential sources of bias and mitigation strategies were considered during study design. As this is a single-arm interventional study, no comparative superiority claims will be made, and findings will be interpreted against published literature. Measurement bias will be minimized using a single masked examiner, averaging 3 keratometry readings, and excluding cases with significant mire distortion. Surgeon learning curve bias will be reduced through prestudy competency requirements, supervised initial cases, and monitoring of incision size. IOL calculation bias will be minimized by using standardized uncorrected keratometry without empirical adjustments, with refractive surprise analyzed as a secondary outcome. Selection bias will be reduced through consecutive recruitment and maintenance of a screening log documenting exclusions and refusals.

### Reporting Guidelines

This protocol was prepared according to the SPIRIT (Standard Protocol Items: Recommendations for Interventional Trials) 2013 checklist for interventional trial protocols. Given the nonrandomized, single-arm design, the TREND (Transparent Reporting of Evaluations With Nonrandomized Designs) statement has also informed the reporting structure.

### Ethical Considerations

This study was approved by the institutional ethics committee of Datta Meghe Institute of Higher Education and Research (Deemed University; DMIHER(DU)/IEC/2025/371) on June 30, 2025, and will be conducted in accordance with the Declaration of Helsinki (2013 revision) and ICMR National Ethical Guidelines for Biomedical and Health Research Involving Human Participants (2017). The study was prospectively registered with the Clinical Trials Registry–India (CTRI/2025/10/095785) before participant enrollment. Written informed consent will be obtained from all participants in their preferred language (English, Hindi, or Marathi) using the participant information sheet (Multimedia Appendix 2) and informed consent form (Multimedia Appendix 3). Participant confidentiality will be maintained through anonymized study codes and secure data storage. As all participants have independent clinical indications for both cataract surgery and pterygium excision, the protocol-defined simultaneous procedure represents standard clinical practice with minimal additional research-related risk, while providing clinically relevant evidence regarding postoperative astigmatism following simultaneous SICS and pterygium surgery.

## Results

Ethics approval (DMIHER(DU)/IEC/2025/371; June 30, 2025) and prospective trial registration with the Clinical Trials Registry–India (CTRI/2025/10/095785; October 9, 2025) were obtained.

Participant recruitment commenced on November 1, 2025, at AVBRH, India, and is ongoing, As of September 2026, 40 participants have been enrolled with data analysis planned after completion of the final follow-up.

## Discussion

### Anticipated Primary Findings

This prospective single-arm interventional study evaluates corneal astigmatism and visual outcomes following simultaneous manual SICS and bare sclera pterygium excision. The central scientific question is the net astigmatic outcome when SICS-induced against-the-rule astigmatism interacts with the reduction in pterygium-induced with-the-rule astigmatism. Alpins vector analysis will assess the vector components of astigmatic change and explore whether net outcomes vary by pterygium size and refractive characteristics.

### Comparison With Prior Work

Previous evidence on simultaneous pterygium and cataract surgery primarily involves phacoemulsification, which generally reduces postoperative astigmatism but may result in less-predictable refractive outcomes in larger pterygia [13,14]. In contrast, manual SICS induces additional against-the-rule astigmatism because of its larger scleral tunnel incision, yet evidence evaluating simultaneous SICS with pterygium excision remains limited despite widespread use in resource-limited settings [14-18]. Pterygium dimensions have been associated with astigmatism and refractive outcomes after cataract surgery [9,21,23,24]. This study addresses these gaps using Alpins vector analysis to characterize net astigmatic change and explore refractive outcomes according to pterygium size [22].

### Strengths and Limitations

Strengths include the prospective design, standardized surgical protocol, masked keratometric assessment, a single-surgeon approach to reduce procedural variability, and use of vector-based astigmatism analysis. Limitations include the absence of a comparator group, the restriction to a single center and surgeon, the exploratory nature of refractive prediction analyses, and the relatively short 30-day follow-up period, which may not capture complete corneal stabilization or permit definitive assessment of pterygium recurrence. The lack of corneal topography limits detailed characterization of irregular astigmatism and higher-order aberrations, although this reflects real-world practice in resource-limited SICS-dominant settings where advanced imaging is not routinely available. The use of manual keratometry, while clinically feasible, may not fully capture the complex corneal surface changes following combined surgery, particularly in eyes with larger pterygia or irregular astigmatism. Findings should therefore be interpreted primarily as short-term postoperative outcomes.

### Future Directions

Future studies should evaluate longer follow-up periods, incorporate corneal topography, compare simultaneous and sequential approaches, and validate refractive prediction models prospectively. Data generated from this study may contribute toward the development of pterygium size–adjusted IOL calculation strategies for simultaneous SICS procedures.

### Dissemination Plan

Findings from this study will be disseminated through (1) publication of the primary results paper in a peer-reviewed international ophthalmology journal, (2) presentation at national and regional ophthalmology conferences and meetings, and (3) publication of a secondary paper reporting the Alpins vector analysis results. No commercial dissemination is intended.

### Conclusions

This protocol provides a rigorous framework for prospective evaluation of corneal astigmatism following simultaneous SICS and bare sclera pterygium excision in a resource-limited setting. Upon completion, the findings may inform surgical planning, IOL power selection, and patient counseling in regions where SICS remains the dominant cataract surgery technique, although these clinical implications will be confirmed only after data analysis.

## Appendix

Multimedia Appendix 1 Data collection form.

Multimedia Appendix 2 Patient information sheet.

Multimedia Appendix 3 Informed consent form.

## Abbreviations

ASSORT: Alpins Statistical System for Ophthalmic Refractive Surgery Techniques
AVBRH: Acharya Vinoba Bhave Rural Hospital
BCVA: best-corrected visual acuity
CRF: case record form
DV: difference vector
IOL: intraocular lens
LOCS-III: Lens Opacities Classification System III
logMAR: logarithm of the minimum angle of resolution
MCID: minimal clinically important difference
MMRM: mixed models for repeated measures
OPD: outpatient department
PMMA: poly (methyl methacrylate)
SIA: surgically induced astigmatism
SICS: small incision cataract surgery
SPIRIT: Standard Protocol Items: Recommendations for Interventional Trials
SRK/T: Sanders Retzlaff Kraff theoretical
TIA: target induced astigmatism vector
TREND: Transparent Reporting of Evaluations With Nonrandomized Designs
UCVA: uncorrected visual acuity

## Data Availability

Deidentified participant-level datasets generated during this study will be made available from the corresponding author upon reasonable request after publication of the primary results paper, subject to applicable institutional data governance policies.
