# Supplementary material for: Corneal Astigmatism Outcomes After Simultaneous Pterygium Excision Using The Bare Sclera Technique and Manual Small Incision Cataract Surgery: Protocol for a Prospective Single-Arm Pre-Post Interventional Cohort Study
Source: JMIR Res Protoc. 2026 Sep 23;15:e96749. doi: 10.2196/96749 (PMC13646781; doi:10.2196/96749)
Supplement: Supplementary file 1 [file resprot_v15i1e96749_app1.docx]

**DATA COLLECTION PROFORMA**

Study Title: *Effect of Simultaneous Pterygium Excision on Corneal Astigmatism Following Manual Small Incision Cataract Surgery*

Study Site: Department of Ophthalmology,
Acharya Vinoba Bhave Rural Hospital
Datta Meghe Institute of Higher Education and Research, Wardha

Principal Investigator: Dr. Akshaya Anant Tandle

Study Code No: __________

1. Patient Identification

Patient Name: ______________________
Hospital Registration Number: __________________
Study ID: __________________

Age: ______ years
Gender: □ Male □ Female □ Other

Address: ___________________________

Mobile Number: _____________________

Eye Included in Study
□ Right Eye
□ Left Eye

Date of Enrollment: ___ / ___ / _____

2. Medical History

History of systemic disease

□ Diabetes Mellitus
□ Hypertension
□ Both
□ None

Duration of symptoms (months/years): __________

History of ocular trauma
□ Yes □ No

History of previous ocular surgery
□ Yes □ No

History of contact lens use
□ Yes □ No

History of chronic eye rubbing
□ Yes □ No

3. Ocular Examination (Preoperative)

Visual Acuity

Uncorrected Visual Acuity (UCVA): __________

Best Corrected Visual Acuity (BCVA): __________

Intraocular Pressure (mmHg): __________

Anterior Segment Examination

Cornea: ___________________
Pterygium Grade: □ Grade 1 □ Grade 2

Pterygium Location
□ Nasal
□ Temporal

Pterygium Size

Horizontal extension onto cornea (mm): ______

Vertical width (mm): ______

Corneal involvement (mm): ______

Lens Status (Cataract Type)

□ Nuclear
□ Cortical
□ Posterior Subcapsular
□ Mixed

Fundus Examination

□ Normal
□ Abnormal (Specify): __________

4. Keratometry (Preoperative)

K1 (Flat Meridian): ______ D

Axis: ______°

K2 (Steep Meridian): ______ D

Axis: ______°

Corneal Astigmatism: ______ D

Type of Astigmatism

□ With-the-Rule
□ Against-the-Rule
□ Oblique

5. Surgical Details

Date of Surgery: ___ / ___ / _____

Type of Surgery

□ Manual Small Incision Cataract Surgery

Pterygium Procedure

□ Excision with Conjunctival Autograft

Surgeon: ____________________

Incision Site

□ Superior
□ Superotemporal

Incision Size (mm): ______

Intraocular Lens Implanted

IOL Type: __________

IOL Power: ______ D

Intraoperative Complications

□ None
□ Yes (Specify): __________

6. Postoperative Evaluation

Day 1

UCVA: ______

Anterior Segment Findings

□ Clear Cornea
□ Edema
□ Conjunctival graft status: __________

Complications: __________________

Day 30

UCVA: ______
BCVA: ______

Keratometry

K1: ______ D

K2: ______ D

Astigmatism: ______ D

Complications: __________________

Day 60

UCVA: ______
BCVA: ______

Keratometry

K1: ______ D

K2: ______ D

Astigmatism: ______ D

Day 90 (Primary Outcome)

UCVA: ______
BCVA: ______

Keratometry

K1: ______ D

K2: ______ D

Axis: ______

Corneal Astigmatism: ______ D

Refractive Error: __________________

7. Surgically Induced Astigmatism (Vector Analysis)

Target Induced Astigmatism (TIA): ______

Surgically Induced Astigmatism (SIA): ______

Difference Vector (DV): ______

Correction Index (CI): ______

Method Used:
□ Alpins Vector Analysis

8. Patient Satisfaction (Day 90)

Visual Satisfaction

□ Very Satisfied
□ Satisfied
□ Neutral
□ Dissatisfied

Glare/Discomfort

□ Yes □ No

Need for Spectacles

□ Yes □ No

Overall Satisfaction Score: ______

9. Postoperative Complications

□ Infection
□ Corneal scarring
□ Recurrence of pterygium
□ Persistent astigmatism
□ IOL related complication
□ None

Specify: _______________________

10. Investigator Remarks

Signature of Investigator: __________

Date: ___ / ___ / _____
