# Supplementary material for: Corneal Astigmatism Outcomes After Simultaneous Pterygium Excision Using The Bare Sclera Technique and Manual Small Incision Cataract Surgery: Protocol for a Prospective Single-Arm Pre-Post Interventional Cohort Study
Source: JMIR Res Protoc. 2026 Sep 23;15:e96749. doi: 10.2196/96749 (PMC13646781; doi:10.2196/96749)
Supplement: Supplementary file 2 [file resprot_v15i1e96749_app2.docx]

**PATIENT INFORMATION SHEET (ENGLISH)**

Study Title:
Corneal Astigmatism Outcomes After Simultaneous Pterygium Excision and Small Incision Cataract Surgery: A Prospective Observational Study

Principal Investigator:
Dr. Akshaya Anant Tandle
Resident, Department of Ophthalmology
Jawaharlal Nehru Medical College, Wardha
Datta Meghe Institute of Higher Education and Research
Sawangi (Meghe), Wardha, Maharashtra, India – 442107

Mobile Number: 9820421538

Study conducted at:
Department of Ophthalmology
Acharya Vinoba Bhave Rural Hospital, Wardha

1. Introduction

You are being invited to participate in a research study conducted at Acharya Vinoba Bhave Rural Hospital. Before you decide whether to participate, it is important for you to understand why the research is being done and what it will involve. Please read the following information carefully.

You may ask any questions if something is not clear.

2. Purpose of the Study

Some patients have both cataract and pterygium (a growth on the white part of the eye extending onto the cornea). Both conditions can reduce vision.

In this study, both problems will be treated in a single surgery. The purpose of the study is to observe how the surgery affects the shape of the cornea and astigmatism (cylindrical power) after the operation.

3. Why Have I Been Asked to Participate?

You have been invited because:

- You are 35 years of age or older
- You have cataract requiring surgery
- You also have a pterygium in the same eye

About 100 patients will participate in this study.

4. Do I Have to Take Part?

No. Participation in this study is completely voluntary.

If you decide not to participate, it will not affect your treatment in any way.
You may also withdraw from the study at any time without giving a reason.

5. What Will Happen If I Take Part?

If you agree to participate:

Before Surgery

You will undergo routine eye examinations including:

- Vision testing
- Measurement of corneal curvature
- Slit-lamp examination
- Eye pressure measurement
- Biometry for lens power calculation

These tests are part of standard cataract evaluation.

During Surgery

You will undergo simultaneous cataract surgery and pterygium removal.
This is a standard surgical procedure routinely performed at this hospital.

After Surgery

You will be asked to return for follow-up visits on:

- Day 1
- Day 30
- Day 60
- Day 90

During these visits, we will measure:

- Vision
- Corneal curvature
- Eye pressure
- Eye health

At the final visit (Day 90), you will also be asked a few questions about your satisfaction with vision after surgery.

6. Are There Any Risks?

The surgery is standard treatment for cataract and pterygium.
The risks are the same as routine eye surgery and may include:

- Eye redness
- Infection
- Swelling
- Temporary vision changes

Your doctor will explain these risks in detail before surgery.

7. Are There Any Benefits?

You may benefit from:

- Improved vision
- Removal of pterygium
- Treatment of cataract in a single procedure

The information from this study may also help doctors improve treatment for future patients.

8. Confidentiality

All information collected during this study will be kept strictly confidential.

Your name will not appear in any reports or publications.
Your data will be identified only by a study code number.

9. Compensation

You will receive standard medical care during the study.
There is no additional payment for participation.

10. Contact Information

If you have any questions regarding this study, please contact:

Principal Investigator

Dr. Akshaya Anant Tandle
Mobile: 9820421538
Department of Ophthalmology
Acharya Vinoba Bhave Rural Hospital
Wardha, Maharashtra

**रोगी सूचना पत्र (HINDI)**

अध्ययन का शीर्षक:
एक साथ प्टेरिजियम हटाने और स्मॉल इन्सीजन कैटरैक्ट सर्जरी के बाद कॉर्नियल एस्टिग्मैटिज़्म के परिणामों का अध्ययन

प्रधान अन्वेषक:
डॉ. अक्षया अनंत टंडले
रेज़िडेंट, नेत्र रोग विभाग
Jawaharlal Nehru Medical College, Wardha
Datta Meghe Institute of Higher Education and Research
सावंगी (मेघे), वर्धा, महाराष्ट्र – 442107

मोबाइल नंबर: 9820421538

अध्ययन स्थल:
नेत्र रोग विभाग
Acharya Vinoba Bhave Rural Hospital, वर्धा

1. परिचय

आपको इस शोध अध्ययन में भाग लेने के लिए आमंत्रित किया गया है। यह अध्ययन Acharya Vinoba Bhave Rural Hospital में किया जा रहा है।

इस अध्ययन में भाग लेने का निर्णय लेने से पहले कृपया नीचे दी गई जानकारी को ध्यान से पढ़ें।

2. अध्ययन का उद्देश्य

कुछ मरीजों में मोतियाबिंद (Cataract) और प्टेरिजियम (Pterygium) दोनों होते हैं। दोनों समस्याएं दृष्टि को प्रभावित कर सकती हैं।

इस अध्ययन का उद्देश्य यह देखना है कि दोनों सर्जरी एक साथ करने पर आंख की सतह और एस्टिग्मैटिज़्म पर क्या प्रभाव पड़ता है।

3. आपको क्यों चुना गया है?

आपको इस अध्ययन में इसलिए शामिल होने के लिए कहा गया है क्योंकि:

- आपकी आयु 35 वर्ष या उससे अधिक है
- आपको मोतियाबिंद है
- उसी आंख में प्टेरिजियम भी है

लगभग 100 मरीजों को इस अध्ययन में शामिल किया जाएगा।

4. क्या अध्ययन में भाग लेना अनिवार्य है?

नहीं। इसमें भाग लेना पूरी तरह आपकी इच्छा पर निर्भर है।

यदि आप भाग नहीं लेना चाहते, तो भी आपकी चिकित्सा पर कोई प्रभाव नहीं पड़ेगा।

5. अध्ययन में क्या किया जाएगा?

सर्जरी से पहले

आपकी आंखों की जांच की जाएगी:

- दृष्टि जांच
- कॉर्निया की जांच
- आंख का दबाव
- अन्य नियमित जांच

सर्जरी के दौरान

आपकी मोतियाबिंद सर्जरी और प्टेरिजियम हटाने की सर्जरी एक साथ की जाएगी।

सर्जरी के बाद

आपको निम्नलिखित दिनों में जांच के लिए आना होगा:

- दिन 1
- दिन 30
- दिन 60
- दिन 90

इन जांचों में आपकी दृष्टि और आंख की स्थिति का मूल्यांकन किया जाएगा।

6. संभावित जोखिम

यह सर्जरी सामान्य उपचार का हिस्सा है। संभावित जोखिम हो सकते हैं:

- आंख में लालिमा
- संक्रमण
- सूजन
- दृष्टि में अस्थायी परिवर्तन

7. संभावित लाभ

- दृष्टि में सुधार
- प्टेरिजियम का हटना
- एक ही सर्जरी में दोनों समस्याओं का उपचार

8. गोपनीयता

आपकी सभी जानकारी गोपनीय रखी जाएगी।

आपका नाम किसी भी रिपोर्ट या प्रकाशन में उपयोग नहीं किया जाएगा।

9. क्षतिपूर्ति

अध्ययन के दौरान आपको मानक चिकित्सा देखभाल प्रदान की जाएगी।
इस अध्ययन में भाग लेने के लिए कोई अतिरिक्त भुगतान नहीं दिया जाएगा।

10. संपर्क जानकारी

यदि आपको इस अध्ययन के संबंध में कोई प्रश्न हो, तो कृपया संपर्क करें:

प्रधान अन्वेषक
डॉ. अक्षया अनंत टंडले
मोबाइल: 9820421538
नेत्र रोग विभाग
Acharya Vinoba Bhave Rural Hospital
वर्धा, महाराष्ट्र

**रुग्ण माहिती पत्र (MARATHI)**

अभ्यासाचे शीर्षक:
प्टेरिजियम काढणे आणि स्मॉल इन्सीजन कॅटरॅक्ट सर्जरी एकत्र केल्यानंतर कॉर्नियल अॅस्टिग्मॅटिझमवरील परिणामांचा अभ्यास

मुख्य संशोधक:
डॉ. अक्षया अनंत टंडले
रेसिडेंट, नेत्ररोग विभाग
Jawaharlal Nehru Medical College, Wardha
Datta Meghe Institute of Higher Education and Research
सावंगी (मेघे), वर्धा, महाराष्ट्र – 442107

मोबाइल क्रमांक: 9820421538

अभ्यासाचे ठिकाण:
नेत्ररोग विभाग
Acharya Vinoba Bhave Rural Hospital, वर्धा

1. परिचय

आपल्याला या संशोधन अभ्यासामध्ये सहभागी होण्यासाठी आमंत्रित केले जात आहे. हा अभ्यास Acharya Vinoba Bhave Rural Hospital येथे करण्यात येत आहे.

कृपया खाली दिलेली माहिती काळजीपूर्वक वाचा.

2. अभ्यासाचा उद्देश

काही रुग्णांमध्ये मोतीबिंदू (Cataract) आणि प्टेरिजियम (Pterygium) दोन्ही समस्या असतात.

या अभ्यासाचा उद्देश म्हणजे दोन्ही शस्त्रक्रिया एकत्र केल्यावर कॉर्नियाच्या आकारावर आणि अॅस्टिग्मॅटिझमवर काय परिणाम होतो हे पाहणे.

3. आपणास का निवडले आहे?

आपणास या अभ्यासात सहभागी होण्यासाठी विचारले आहे कारण:

- आपले वय 35 वर्षे किंवा त्यापेक्षा जास्त आहे
- आपल्याला मोतीबिंदू आहे
- त्याच डोळ्यात प्टेरिजियम आहे

या अभ्यासात सुमारे 100 रुग्णांचा समावेश केला जाईल.

4. सहभाग ऐच्छिक आहे का?

होय. या अभ्यासात सहभागी होणे पूर्णपणे ऐच्छिक आहे.

आपण सहभागी न झाल्यास आपल्या उपचारांवर कोणताही परिणाम होणार नाही.

5. अभ्यासात काय केले जाईल?

शस्त्रक्रियेपूर्वी

डोळ्यांची नियमित तपासणी केली जाईल:

- दृष्टी तपासणी
- कॉर्नियाची तपासणी
- डोळ्याचा दाब
- इतर आवश्यक तपासण्या

शस्त्रक्रिया

मोतीबिंदू शस्त्रक्रिया आणि प्टेरिजियम काढण्याची शस्त्रक्रिया एकत्र केली जाईल.

शस्त्रक्रियेनंतर

आपल्याला खालील दिवशी तपासणीसाठी येणे आवश्यक आहे:

- दिवस 1
- दिवस 30
- दिवस 60
- दिवस 90

6. संभाव्य धोके

ही शस्त्रक्रिया नियमित उपचाराचा भाग आहे. संभाव्य धोके:

- डोळा लाल होणे
- संसर्ग
- सूज
- तात्पुरते दृष्टी बदल

7. संभाव्य फायदे

1. दृष्टी सुधारणा
2. प्टेरिजियम काढणे
3. एकाच शस्त्रक्रियेत दोन्ही समस्यांचे उपचार

8. गोपनीयता

या अभ्यासादरम्यान गोळा केलेली सर्व माहिती पूर्णपणे गोपनीय ठेवली जाईल.
आपले नाव कोणत्याही अहवालात किंवा प्रकाशनात नमूद केले जाणार नाही.
आपली माहिती केवळ अभ्यासाच्या कोड क्रमांकाद्वारे ओळखली जाईल.

9. भरपाई

अभ्यासादरम्यान आपल्याला नियमित वैद्यकीय सेवा दिली जाईल.
या अभ्यासात सहभागी झाल्याबद्दल कोणतेही अतिरिक्त मानधन दिले जाणार नाही.

10. संपर्क माहिती

या अभ्यासाबाबत काही प्रश्न असल्यास कृपया संपर्क साधा:

मुख्य संशोधक
डॉ. अक्षया अनंत टंडले
मोबाइल: 9820421538
नेत्ररोग विभाग
Acharya Vinoba Bhave Rural Hospital
वर्धा, महाराष्ट्र
