# Supplementary material for: Corneal Astigmatism Outcomes After Simultaneous Pterygium Excision Using The Bare Sclera Technique and Manual Small Incision Cataract Surgery: Protocol for a Prospective Single-Arm Pre-Post Interventional Cohort Study
Source: JMIR Res Protoc. 2026 Sep 23;15:e96749. doi: 10.2196/96749 (PMC13646781; doi:10.2196/96749)
Supplement: Supplementary file 3 [file resprot_v15i1e96749_app3.docx]

INFORMED CONSENT FORM (ENGLISH)

Title of Study:
Effect of Simultaneous Pterygium Excision on Corneal Astigmatism Following Manual Small Incision Cataract Surgery

Principal Investigator:
Dr. Akshaya Anant Tandle
Resident, Department of Ophthalmology
Jawaharlal Nehru Medical College, Wardha
Datta Meghe Institute of Higher Education and Research
Sawangi (Meghe), Wardha, Maharashtra, India – 442107

Mobile Number: 9820421538

Study conducted at:
Department of Ophthalmology
Acharya Vinoba Bhave Rural Hospital, Wardha

1. Introduction

You are being invited to participate in a research study conducted by the Department of Ophthalmology at Acharya Vinoba Bhave Rural Hospital. Before deciding whether to participate, it is important that you understand the purpose of the study and what it involves. Please read the information carefully. You may ask any questions.

Participation in this study is completely voluntary.

2. Purpose of the Study

This study aims to evaluate changes in corneal astigmatism after simultaneous pterygium removal and cataract surgery.

The results may help improve surgical planning and visual outcomes for patients with cataract and pterygium.

3. Procedures

If you agree to participate:

- Your eye will be examined in detail.
- Measurements of vision and corneal curvature will be taken.
- Cataract surgery along with removal of pterygium will be performed.
- You will be followed up after surgery on:
  - Day 1
  - Day 30
  - Day 60
  - Day 90

Each visit will include routine eye examinations.

4. Possible Benefits

You may benefit from:

- Removal of cataract and pterygium
- Improvement in vision

The information from this study may benefit future patients.

5. Possible Risks or Discomfort

As with any eye surgery, there may be risks including:

- Infection
- Redness or irritation
- Astigmatism
- Delayed healing

All procedures will be performed by qualified ophthalmic surgeons.

6. Confidentiality

All personal information will remain confidential. Your identity will not be revealed in any report or publication.

7. Voluntary Participation

Your participation is voluntary.

You are free to withdraw from the study at any time without affecting your medical care.

8. Contact Information

If you have any questions about this study, please contact:

Principal Investigator:

Dr. Akshaya Anant Tandle
Mobile: 9820421538
Department of Ophthalmology
Acharya Vinoba Bhave Rural Hospital, Wardha

9. Consent Statement

I have read and understood the information provided above. I have had the opportunity to ask questions and received satisfactory answers.

I voluntarily agree to participate in this study.

Participant Name: ___________________

Signature / Thumb Impression: ___________________

Date: ___________________

Investigator Signature: ___________________

सहमति पत्र (HINDI)

अध्ययन का शीर्षक:
प्टेरीजियम हटाने के साथ मोतियाबिंद सर्जरी के बाद कॉर्नियल एस्टिग्मैटिज़्म में होने वाले परिवर्तन का अध्ययन

प्रधान अन्वेषक:
डॉ. अक्षया अनंत टंडले
रेज़िडेंट, नेत्र रोग विभाग
Jawaharlal Nehru Medical College, Wardha
Datta Meghe Institute of Higher Education and Research
सावंगी (मेघे), वर्धा, महाराष्ट्र – 442107

मोबाइल नंबर: 9820421538

अध्ययन स्थल:
नेत्र रोग विभाग
Acharya Vinoba Bhave Rural Hospital, वर्धा

1. परिचय

आपको इस शोध अध्ययन में भाग लेने के लिए आमंत्रित किया जा रहा है। यह अध्ययन नेत्र रोग विभाग द्वारा किया जा रहा है।

इस अध्ययन में भाग लेना पूरी तरह से स्वैच्छिक है।

2. अध्ययन का उद्देश्य

इस अध्ययन का उद्देश्य प्टेरीजियम हटाने और मोतियाबिंद सर्जरी एक साथ करने के बाद कॉर्निया में होने वाले एस्टिग्मैटिज़्म के परिवर्तन का अध्ययन करना है।

3. प्रक्रिया

यदि आप इस अध्ययन में भाग लेने के लिए सहमत होते हैं:

- आपकी आंखों की पूरी जांच की जाएगी।
- दृष्टि और कॉर्निया की वक्रता की माप ली जाएगी।
- मोतियाबिंद सर्जरी के साथ प्टेरीजियम हटाया जाएगा।
- ऑपरेशन के बाद निम्नलिखित दिनों पर जांच की जाएगी:

दिन 1
दिन 30
दिन 60
दिन 90

4. संभावित लाभ

- मोतियाबिंद और प्टेरीजियम का उपचार
- दृष्टि में सुधार

5. संभावित जोखिम

सर्जरी के साथ कुछ जोखिम हो सकते हैं जैसे:

- संक्रमण
- आंखों में लालिमा
- एस्टिग्मैटिज़्म
- घाव भरने में देरी

6. गोपनीयता

आपकी व्यक्तिगत जानकारी पूरी तरह गोपनीय रखी जाएगी।

7. स्वैच्छिक भागीदारी

इस अध्ययन में भाग लेना आपकी इच्छा पर निर्भर है। आप किसी भी समय अध्ययन से बाहर हो सकते हैं।

8. संपर्क जानकारी

यदि आपको अध्ययन के बारे में कोई प्रश्न हो, तो आप संपर्क कर सकते हैं:

डॉ. अक्षया अनंत टंडले
मोबाइल: 9820421538

9. सहमति

मैंने इस अध्ययन के बारे में दी गई जानकारी पढ़ी और समझी है। मुझे प्रश्न पूछने का अवसर मिला।

मैं स्वेच्छा से इस अध्ययन में भाग लेने के लिए सहमत हूँ।

प्रतिभागी का नाम: ____________

हस्ताक्षर / अंगूठा निशान: ____________

दिनांक: ____________

संमती पत्र (MARATHI)

अभ्यासाचे शीर्षक:
मोतीबिंदू शस्त्रक्रियेसोबत प्टेरीजियम काढल्यानंतर कॉर्नियल अॅस्टिग्मॅटिझममधील बदलांचा अभ्यास

मुख्य संशोधक:
डॉ. अक्षया अनंत टंडले
रेसिडेंट, नेत्ररोग विभाग
Jawaharlal Nehru Medical College, Wardha
Datta Meghe Institute of Higher Education and Research
सावंगी (मेघे), वर्धा, महाराष्ट्र – 442107

मोबाइल क्रमांक: 9820421538

अभ्यासाचे ठिकाण:
नेत्ररोग विभाग
Acharya Vinoba Bhave Rural Hospital, वर्धा

1. परिचय

आपल्याला या संशोधन अभ्यासामध्ये सहभागी होण्यासाठी आमंत्रित केले जात आहे. हा अभ्यास नेत्ररोग विभागामार्फत केला जात आहे.

या अभ्यासामध्ये सहभागी होणे पूर्णपणे स्वेच्छेने आहे.

2. अभ्यासाचा उद्देश

प्टेरीजियम काढणे आणि मोतीबिंदू शस्त्रक्रिया एकाच वेळी केल्यानंतर कॉर्नियामधील अॅस्टिग्मॅटिझममधील बदलांचा अभ्यास करणे हा या संशोधनाचा उद्देश आहे.

3. प्रक्रिया

आपण सहभागी होण्यास सहमत असल्यास:

- आपल्या डोळ्यांची तपासणी केली जाईल.
- दृष्टी आणि कॉर्नियाची वक्रता मोजली जाईल.
- मोतीबिंदू शस्त्रक्रियेसोबत प्टेरीजियम काढले जाईल.
- शस्त्रक्रियेनंतर खालील दिवशी तपासणी होईल:

दिवस 1
दिवस 30
दिवस 60
दिवस 90

4. संभाव्य फायदे

- मोतीबिंदू आणि प्टेरीजियमचा उपचार
- दृष्टी सुधारणा

5. संभाव्य धोके

शस्त्रक्रियेमध्ये काही धोके असू शकतात:

- संसर्ग
- डोळा लाल होणे
- अॅस्टिग्मॅटिझम
- जखम भरून येण्यास विलंब

6. गोपनीयता

आपली वैयक्तिक माहिती पूर्णपणे गोपनीय ठेवली जाईल.

7. स्वेच्छेने सहभाग

आपला सहभाग पूर्णपणे स्वेच्छेने आहे. आपण कोणत्याही वेळी अभ्यासातून बाहेर पडू शकता.

8. संपर्क माहिती

अभ्यासाबाबत काही शंका असल्यास कृपया संपर्क साधा:

डॉ. अक्षया अनंत टंडले
मोबाइल: 9820421538

9. संमती

मी वरील माहिती वाचली व समजून घेतली आहे आणि मी या अभ्यासात सहभागी होण्यास सहमत आहे.

सहभागीचे नाव: ____________

स्वाक्षरी / अंगठा ठसा: ____________

दिनांक: ____________
